# Female hormones enhance skin T cell expansion and suppress IFN-γ production

**DOI:** 10.3389/fimmu.2026.1903552

**Published:** 2026-10-01

**Authors:** Rajia Bahri, Chiara Tontini, Orsolya Kiss, Abigail K. Langton, Victoria L. Newton, Mike Bell, Silvia Bulfone-Paus

**Affiliations:** 1 Lydia Becker Institute of Immunology, School of Biological Sciences, Faculty of Biology Medicine and Health, University of Manchester, Manchester, United Kingdom; 2 Centre for Dermatology Research, Division of Musculoskeletal and Dermatological Sciences, School of Biological Sciences, The University of Manchester and Salford Royal NHS Foundation Trust, Manchester Academic Health Science Centre, Manchester, United Kingdom; 3 No7 Beauty Company, The Boots Group, Nottingham, United Kingdom

**Keywords:** estrogen receptor, hormone replacement therapy, menopause, peripheral blood mononuclear cells, sodium lauryl sulfate, skin immune response

## Abstract

**Introduction:**

Estrogen receptors (ERs) regulate immune function, but their expression across immune cell subsets and hormone responsiveness in human skin are not fully understood.

**Methods:**

We used 10-color flow cytometry to quantify ER (ERα, ERβ, and GPER1) expression in peripheral blood mononuclear cells (PBMCs) from healthy donors. We analyzed sex hormone gene expression using public single-cell RNA datasets. Skin immune responses to sodium lauryl sulfate (SLS) were assessed in pre- and post-menopausal women, with or without hormone replacement therapy (HRT). Furthermore, we measured the production of mediators in the ER expressing immune cytotoxic cells, specifically T and NK cells, in response to pre- and post-menopausal hormone cocktails.

**Results:**

ERα was predominantly expressed on the membrane of NK and NKT cells, while ERβ was mainly intracellular and showed low expression across subsets. GPER1 displayed a similar membrane distribution to ERα but at lower expression levels. T cells displayed moderate membrane ERα and lower GPER1 expression. Gene expression analysis in women revealed strong ESR1 and ESR2 expression in T cells. In inflamed skin, HRT selectively increased T cell numbers after SLS exposure in post-menopausal women. *In vitro*, female hormones dose-dependently decreased IFN-γ production in T and NK cells but did not alter cytotoxic mediator production in these cells.

**Conclusions:**

ER expression levels and localization vary by immune cell subset, indicating that estrogen modulates immune responses in a cell-specific manner. Sex hormones differentially impact T and NK cell populations during skin inflammation, by increasing T cell numbers and, reducing IFN-γ, without altering cytotoxic activity. These findings demonstrate that female sex hormones significantly influence immune responses in inflamed and aging skin and may guide the development of targeted therapeutic strategies.

## Introduction

Estrogens regulate immune function by interacting with signaling pathways that control inflammation and immune responses. Through their receptors, they influence innate immunity by affecting macrophages, dendritic cells (DCs), and natural killer (NK) cells, and modulate adaptive immunity by acting on T and B lymphocytes (1–3). Estrogen promotes alternative macrophage activation via ERα signaling (4, 5), stimulates DC differentiation and antigen-presenting functions via ERα (6), reduces NK cell cytotoxicity and alters cytokine production through ERα and ERβ (7, 8). It also regulates T cell activation, differentiation, and cytokine production primarily via estrogen receptors (9, 10), and controls B cell survival, activation, differentiation, and antibody production (11, 12). These mechanisms contribute to sex-biased patterns in inflammatory skin diseases such as atopic dermatitis, which often vary with the menstrual cycle, pregnancy, and menopause (13, 14).

Declining estrogen levels, as observed during peri- and post-menopause, reduce estrogen receptor-mediated immune signaling, potentially leading to altered immune responses, increased susceptibility to inflammation, and impaired tissue repair (15, 16). This transition is marked by changes in the Th1/Th17/Treg balance and NK cell proportions (17, 18). In post-menopausal women, hormone replacement therapy (HRT) lowers elevated plasma levels of the pro-inflammatory cytokines TNF-α, IFN-γ, and IL-6 (16, 19, 20).

In our previous study, post-menopausal women receiving HRT show improved skin barrier responses to sodium lauryl sulfate (SLS) challenge, accompanied by increased numbers of Langerhans cells, inflammatory DCs, and macrophages in the dermis (21), suggesting that estrogens modulate immune cell recruitment and activation during skin barrier disruption. However, the direct impact of estrogens on human cutaneous cytotoxic immune processes remains poorly understood, even though cytotoxic immune subsets such as T cells and NK cells are known to play a role in the skin immune response (22, 23).

This study investigates hormone-immune cell interactions and aims to clarify how female sex hormones modulate cytotoxic immune cells in the context of skin inflammation. First, we provide a comprehensive characterization of ER expression in both the membrane-only and combined membrane and intracellular compartments across major human immune cell populations to identify those most influenced by estrogens. Second, building on our previous research on SLS-induced barrier disruption (21), we analyze cytotoxic immune cell subsets in SLS-challenged skin from healthy pre-menopausal women, post-menopausal women, and post-menopausal women receiving HRT. Finally, we explore *in vitro* how female sex hormones affect the activity of selected cytotoxic immune cell populations. Our results demonstrate that female sex hormones enhance T cell numbers in inflamed skin and selectively reduce IFN-γ production in T and NK cells without altering cytotoxic mediators. These findings highlight a critical role for estrogens in modulating immune responses during skin inflammation.

## Materials and methods

### PBMCs

Peripheral blood leukocyte cones from healthy anonymous donors of both genders (Manchester NHS Blood & Transplant Clinic) were used. PBMCs were isolated by density gradient centrifugation (Ficoll-Paque, Cytiva) as previously described (24) and frozen in 10% DMSO in fetal bovine serum (FBS).

### Multiparametric flow cytometry analysis of ERs

After thawing, PBMCs were first washed in pre-warmed RPMI (Sigma) containing 10% FBS, 10% Penicillin-Streptomycin, and 2 mM L-glutamine. Cells were then prepared for staining. Unstimulated PBMCs were stained for surface and intracellular markers using antibodies (see **Supplementary Table S2**). For surface staining, cells were blocked with Human True StainFcX (BioLegend), stained with specific markers (CD45, CD3, CD20, CD56, CD14, CD16, HLA-DR, ERα, ERβ and GPER1) for 30 min at 4 °C, then live/dead-stained (Thermo Fisher Scientific), and fixed in 4% formaldehyde for 20 min at 4 °C.

To assess the total expression of ER, following surface and live/dead staining, cells were fixed and permeabilized with 1x buffer (Invitrogen). Subsequently, intracellular staining for estrogen receptors (ERα, ERβ, GPER1, same antibodies as used for surface staining) was performed for 1 h at room temperature. Cells were then washed, resuspended in FACS buffer, and analyzed on a Symphony flow cytometer (BD Biosciences).

### Flow cytometry data analysis

Flow cytometry files were compensated with UltraComp Beads (Invitrogen) and processed in FlowJo (BD Biosciences). Debris, non-viable cells, and aggregates were removed (**Supplementary Figure 1A**) before manual and unsupervised clustering. Manual gating in FlowJo identified immune cell populations (see **Supplementary Figures 1A, B**). Positive gates were set using Fluorescence Minus One (FMO) controls, in which the fluorochrome for the marker of interest was omitted from an otherwise complete staining panel, allowing accurate discrimination of positive and negative populations. Marker expression was analyzed and plotted with Prism 10 (GraphPad).

### Unsupervised analysis workflow

Live single CD45+ cells from each sample were exported from FlowJo. Combined FCS files were uploaded to OMIQ (Dotmatics). Custom scaling co-factors for individual markers were set based on fluorescence spread to optimize data visualization, and fluorescence values were converted using the inverse hyperbolic sine (asinh) transformation. Batch correction was performed using CyCombine (25) to remove technical variation between different experimental rounds. Dimensionality reduction was performed using Uniform Manifold Approximation and Projection (UMAP) to visualize immune cell subpopulations in two-dimensional space. Unbiased clustering was performed using FlowSOM (10.1002/cyto.a.22625). Cell populations were assigned using marker expression heatmaps. Similar clusters were merged based on marker expression to generate final cell subsets. Raw counts and meta-cluster frequencies were exported from OMIQ for downstream statistical analysis.

### Preparation and composition of the hormone cocktails

Estradiol (E887-250MG), Progesterone (5341-25GM), and DHEA (dehydroepiandrosterone, 252805-10GM, all Merck Millipore, Gillingham, UK) were dissolved in sterile, 0.22 µm-filtered ethanol. To prepare the pre-menopause cocktail, 10 mM estradiol, 10 mM progesterone, and 20 mM DHEA were mixed in 1 ml of ethanol. The cocktail was then added to cells to achieve final concentrations of 0.75 nM estradiol, 60 nM progesterone, and 20 nM DHEA. The 10x cocktail was prepared at 10 times the concentration. For the post-menopause cocktail, estradiol, progesterone, and DHEA were used at 0.6 nM, 6 nM, and 2 nM, respectively. These concentrations reflect physiologically relevant female circulating hormone levels, commonly employed in *in vitro* experiments to simulate normal human hormonal conditions, as detailed in a previous study (26).

### PBMC stimulation

For stimulation, thawed cells were washed with RPMI-1640 (10% FBS, 1% penicillin-streptomycin) and counted with trypan blue. 0.4 × 10^6 cells/well were plated in 96-well plates and treated with post-menopause, pre-menopause, 10x pre-menopause cocktail, or media.

After the cells rested for 10 min at room temperature, they were stimulated with LPS (100 ng/mL) or left unstimulated as a control. The cells were incubated at 37 °C for 18–20 h. After incubation, Brefeldin A (1:1000) was first added to the cells. This was followed by the addition of PMA (100 nM) and Ionomycin (1 μg/mL).

Cells were rested for 5 h at 37 °C before viability staining with UV zombie dye (1:1000). CD11b membrane staining was performed, followed by fixation (4% formaldehyde, 20 min, 4 °C) and permeabilization (10% buffer) for intracellular staining. Antibodies against Granzyme B, perforin, IFN-γ, CD56, and CD3 were applied for 45–60 min at 4 °C. After incubation, cells were washed and stored at 4 °C until acquisition on a BD Symphony flow cytometer.

### Participant study

We analyzed previously collected skin biopsy samples from participants enrolled in the study by *Kiss* et al. (21). Clinical research was held at the Salford Royal NHS Foundation Trust between December 2017–April 2018. Enrolled subjects included 8 pre-menopausal women (pre-M) aged 39-46, 16 post-menopausal women (9 post-M and 7 post-M+HRT groups), aged 48–63, without menstruation ≥1 year. Exclusion criteria were the use of oral contraceptives or a positive history of psoriasis/eczema. Menopausal status was self-reported and confirmed by serum estrogen and FSH dosing. HRT+ were women on combined or estrogen-only therapy, confirmed by increased estrogen serum levels (see **Table 1**). All were healthy participants of white Northern European ethnicity. Skin was sampled from sun-protected buttock areas.

**Table 1:** Participant characteristics.

Participant number	Age (years)	Menopause status	Estradiol (pmol/l)	FSH (U/l)	SLS-challenge score
1	40	pre-M	1117	17.6	2
2	43	pre-M	446	4.8	1
3	40	pre-M	466	2.4	0
4	48	pre-M	341	4.4	0
5	42	pre-M	336	10.2	1
6	39	pre-M	238	3.9	0
7	48	pre-M	100	5.9	1
8	46	pre-M	310	2.5	2
9	63	post-M+HRT	215	57	1
10	48	post-M+HRT	222	13	1
11	49	post-M+HRT	353	40.1	0
12	56	post-M+HRT	297	53.8	1
13	51	post-M+HRT	314	23.5	0
14	62	post-M+HRT	154	53.7	1
15	55	post-M+HRT	145	20.1	1
16	60	post-M	73	37.9	1
17	63	post-M	<44	92.3	1
18	62	post-M	<44	73.3	1
19	48	post-M	56	63.3	2
20	59	post-M	61	51.1	1
21	53	post-M	87	61	2
22	57	post-M	86	45	1
23	58	post-M	50	91	0
24	49	post-M	64	62	0

Pre-M, pre-menopause; post-M, post-menopause; HRT, hormonal replacement therapy.

### Application of sodium lauryl sulfate patches and clinical assessment

Sodium lauryl sulfate (SLS, 1.25% w/v, Merck) was applied to sun-protected buttock skin using a Finn chamber (20 μL on an 8-mm disc) for 48 hours.

### Skin sampling and histological analysis

After clinical assessment, 6-mm punch biopsies were taken from SLS-challenged and control areas under local anesthesia (2% lignocaine). Each sample was bisected. One-half was fixed in neutral buffered formalin, paraffin-embedded (FFPE), and sectioned at 5 μm.

### Skin immunofluorescence staining

FFPE skin sections were deparaffinized, rehydrated, and boiled in Tris-EDTA buffer (pH 9.0, Abcam) at 95 °C for 30 min for antigen retrieval. Expression of CD3, CD56, and ERα was determined by immunofluorescence staining (see antibody details, **Supplementary Table S2**). Sections were blocked (5% donkey serum) and incubated overnight at 4 °C with primary antibodies (CD3, CD56, ERα). Sections were visualized with AF488, AF555, and AF647 and incubated for 2 h at room temperature. All sections were mounted in Fluoromount-G with DAPI before image analysis. Images were captured on an Olympus BX53 microscope with a 40× objective, DP75 camera, and CellSens software, using filter sets to prevent channel bleedthrough. For each individual, nine non-overlapping images from three skin sections were analyzed with ImageJ. Cell subpopulations were quantified in QuPath. Regions of interest (ROIs) were first defined under the epidermis. Cell detection in QuPath used standard parameters. Subpopulations were manually annotated, then a classifier was trained for each donor and applied to all images. Cell counts per population were exported to Excel for analysis.

### Re-analysis of single-cell RNAseq public datasets

We re-analyzed publicly available scRNAseq datasets from the Sound of Life Cohort (27), accessible at https://apps.allenimmunology.org/aifi/insights/dynamics-imm-health-age/downloads. The datasets included both single cell and pseudobulk scRNAseq counts from PBMC subpopulations obtained from young and elderly cohorts. For this study, we analyzed both the full cohort (n=96, n=55 female, n=41 male, n=49 young, n=47 elderly; 44.52 mean age, range: 25–65 years) and female only participants [n=28 young, n=27 elderly; young aged range: 25–36 years, presumed pre-menopausal and elderly age range: 55–63 years, presumed post-menopausal (28, 29)] to analyze differential gene expression across five immune cell subsets (B cells, NK cells, T cells, monocytes, dendritic cells - DCs). Samples with fewer than 50 cells were excluded from the analysis. Within each cell type, genes were retained only if they had at least 10 counts in a minimum of 3 samples. Differential gene expression between cell subsets was analyzed using DESeq2 (30). Raw pseudobulk counts were normalized using DESeq2’s median-of-ratios method. Targeted gene analyses were performed by assessing the expression of hormone receptors (estrogen receptors: ESR1, ESR2, GPER1) across cell types. For differential analyses, an adjusted p-value < 0.05 (Benjamini-Hochberg correction, B-H) and |Log2 fold change| > 1 was considered statistically significant.

For visualization of cell type structure and estrogen receptor expression at single-cell resolution, up to 500 cells per major cell type were subsampled from the single-cell dataset, log-normalized, and subjected to principal component analysis (30 components) and Uniform Manifold Approximation and Projection (UMAP) embedding using Seurat (31). Cell type identity and expression of ESR1, ESR2, and GPER1 were visualized on the resulting UMAP using ggplot2 (32).

### Statistics

Graphing and analyses were performed in Prism 10.2.2 (GraphPad). Group differences were analyzed using one- or two-way ANOVA with Tukey’s multiple comparisons.

## Results

### Estrogen receptor profiles across peripheral immune cell subsets

The examination of estrogen receptor expression in peripheral blood is crucial for identifying immune cell populations with increased sensitivity to estrogen. Several studies have quantified ERα and ERβ expression in peripheral blood immune cells (33–36). However, data on G protein–coupled estrogen receptor 1 (GPER1), which mediates rapid signaling, remain limited. Inclusion of GPER1 offers a more comprehensive understanding of estrogen biology beyond ERα/ERβ alone.

Few studies have compared estrogen receptor protein expression between intracellular and membrane receptors across immune subsets. To address this gap, PBMCs from six healthy volunteers were analyzed using a 10-color flow cytometry panel to define lymphocyte subsets and quantify ER expression at the plasma membrane and intracellular compartment.

We used supervised (**Supplementary Figure S1**) and unsupervised (**Supplementary Figure S2**) analyses to identify six major immune cell subsets. Both methods produced similar abundance estimates: B cells (supervised: 13-±-2%, unsupervised: 12-±-4%), T cells (supervised: 52-±-7%, unsupervised: 53-±-4%), NKT cells (supervised: 3.6-±-3.3%, unsupervised: 5-±-3%), NK cells (supervised: 2-±-1.3%, unsupervised: 2.5-±-2%), DCs (supervised: 2.5-±-1.5%, unsupervised: 4-±-2.5%) (**Supplementary Figures S1B, A**, **S2E**), and monocytes (supervised: 17.3-±-8%, unsupervised: 15-±-8.5%). These levels align with previously published data (37).

Cell-surface quantification of estrogen receptors showed that ERα is the predominant membrane receptor compared with the other ERs measured by supervised flow analysis. The percentage of cells expressing ERα at the membrane was highest in NKT (95.7 ± 7.5%) and NK (80.2 ± 15.83%) cells. High expression levels were also found in T cells (51.7 ± 11%), B cells (49.2 ± 21.7%), monocytes (45.9 ± 20.3%), and DCs (37.8 ± 9.7%) (**Figures 1A, B**). Total ERα expression, including both intracellular and membrane staining, was high across all cell types, with positivity above 98% (**Figures 1A, B**). The highest intensity was seen in monocytes (**Figure 1C**). Examining the ratio of membrane to total ERα staining, NKT and NK cells exhibited the highest percentage of membrane ERα expression, accounting for nearly all the total expression (95.8% for NKT cells and 80.8% for NK cells), indicating a predominant cell-surface localization in these populations (**Figure 1D**), followed by T cells (52.2%), B cells (49.8%), monocytes (45.9%) and DCs (37.9%) (**Figure 1D**). Geometric mean fluorescence intensity (GMFI) values from supervised analyses were consistent with the median from unsupervised ERα analyses (**Figure 1C**; **Supplementary Figure 2F**).

**Figure 1:**
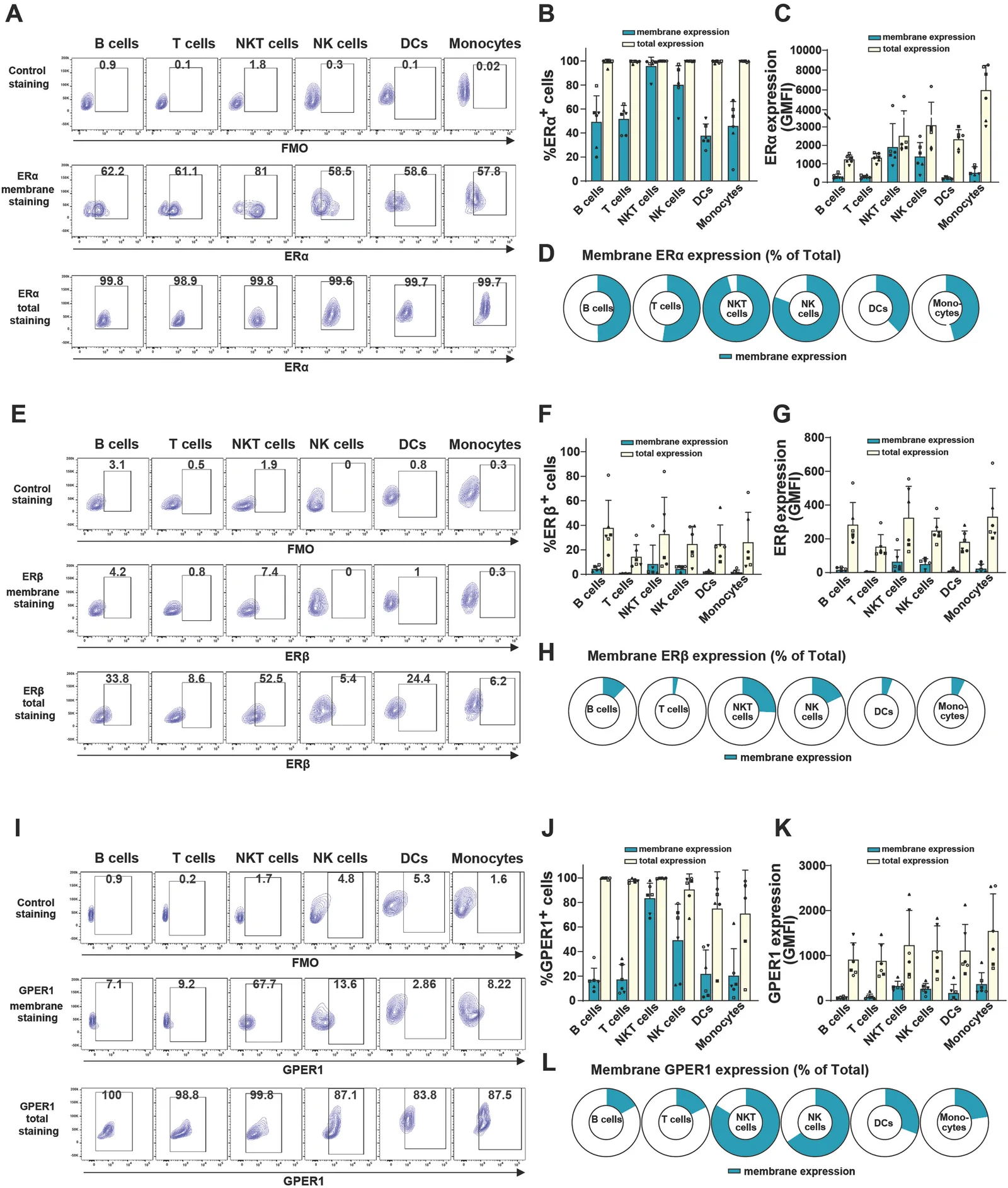
Estrogen receptor expression in PBMCs. Flow cytometry was used to assess estrogen receptor (ER) expression across PBMC immune subsets, including B cells, T cells, natural killer T (NKT) cells, natural killer (NK) cells, dendritic cells (DCs), and monocytes. ER expression was quantified at the cell surface only (membrane expression; dark green bars) and across both membrane and intracellular compartments (total expression; light yellow bars). Panel **(A)** shows representative staining for membrane and total ERα expression, with gates defined using fluorescence minus one (FMO) controls (control staining). Panels **(B, C)** quantify ERα expression using supervised gating, showing the percentage of ERα positive cells and the geometric mean fluorescence intensity (GMFI), respectively. Panel **(D)** shows membrane ERα expression as a percentage of total ERα expression. Panel **(E)** shows representative ERβ staining and its corresponding FMO control. Panels **(F, G)** quantify ERβ expression, showing the percentage of ERβ positive cells and the GMFI, respectively. Panel **(H)** shows membrane ERβ expression as a percentage of total ERβ expression (membrane expression; dark green). Panel **(I)** shows representative GPER1 staining and its corresponding FMO control. Panels **(J**, **K)** quantify GPER1 expression, showing the percentage of GPER1 positive cells and the GMFI, respectively. Panel **(L)** shows membrane GPER1 expression as a percentage of total GPER1 expression (membrane expression; dark green). Data are presented as mean ± standard deviation (n = 6).

In contrast, ERβ was rarely detected on immune cell surfaces and was mainly intracellular. Overall ERβ expression was low (**Figures 1E–H**), with total levels as follows: B cells (supervised: 37.9-±-22.3%), T cells (supervised: 14.4-±-9.7%), NKT cells (supervised: 32.8-±-29.9%), NK cells (supervised: 24.8-±-14.2%), DCs (supervised: 24.7-±-15.5%), and monocytes (supervised: 26.2-±-24.28%) (**Figures 1E, F**). Furthermore, the membrane ERβ expression constitutes a very low percentage of the total expression in all immune subsets, suggesting localization within the intracellular compartment (**Figure 1H**). GMFI data supported these findings (**Figure 1G**). However, the unsupervised median fluorescence indicated higher ERβ membrane expression than the supervised analysis (**Supplementary Figure S2G**). GPER1 expression closely mirrored ERα (**Figures 1I–L**), but at lower membrane levels. The highest membrane GPER1 expression was in NKT cells (83.5 ± 12.1%) and NK cells (49.2 ± 29.4%) followed by DCs (21 ± 19.4%), monocytes (20.3 ± 21.8%), T cells (17.2 ± 12%), and B cells (16.9 ± 9.5%) (**Figure 1J**). The analysis of cell-surface to total GEPR1 staining ratio shows that membrane GPER1 expression in NK and NKT cells constitutes a high percentage of the total GPER1 staining, with 84% for NKT cells and 66% for NK cells (**Figure 1L**). In contrast for other cell types, the ratio of GPER1 membrane staining was 30.7% for DCs, 22.5% for monocytes, 17.7% for T cells and 17% for B cells suggesting that most of the GEPR1 expression occurs in intracellular compartments. (**Figure 1L**). In supervised analysis, intracellular fluorescence was higher in all subsets, whereas in unsupervised analysis, membrane intensity was greater in myeloid cells (**Figure 1K**; **Supplementary Figure 2H**).

Building on these results, we next assessed ER gene expression and reanalyzed an existing dataset (27) to evaluate ER gene expression across various PBMC subsets within a human cohort, irrespective of age and gender. Our analysis revealed that the ESR1 gene, which encodes ERα, has the highest normalized count in T cells, followed by B cells, NK cells, DCs, and monocytes (**Figures 2A, B, E**). ESR2 gene expression, which encodes ERβ, is more pronounced in NK cells, followed by B cells, T cells, and DCs (**Figure 2A, C, E**). In contrast, GPER1 gene expression was detected in DCs, monocytes and, to a lesser extent, B cells (**Figures 2A, D, E**). Notably, gene expression of the receptor for the other female sex hormone, progesterone, typically included in HRT, was absent in PBMCs. To further explore the estrogen receptor expression in immune cells in women, we analyzed gene expression differences between young (25–36 years, presumed premenopausal) and elderly (55–63 years, presumed postmenopausal) women. We used the same publicly available single-cell RNA sequencing dataset of PBMCs as in the previous analysis (27), but this time focusing on females and segmenting by age. As shown in **Figure 2F**, T cells in women exhibit strong expression of ESR1 (ERα) and ESR2 (ERβ) transcripts compared to the other immune cell subsets. Although these genes tend to increase with age in T cells, this trend does not reach statistical significance. In contrast, DCs and monocytes show the lowest levels of ESR2 expression. However, these two cell subsets exhibit the highest GPER1 expression, particularly monocytes, which show strong expression levels. While GPER1 expression in monocytes decreases with age in women, it is not statistically significant, unlike the significant reduction observed in GPER1 expression in T cells. Additionally, NK cells do not seem to express GPER1 in women (**Figure 2F**; **Supplementary Table S1**).

**Figure 2:**
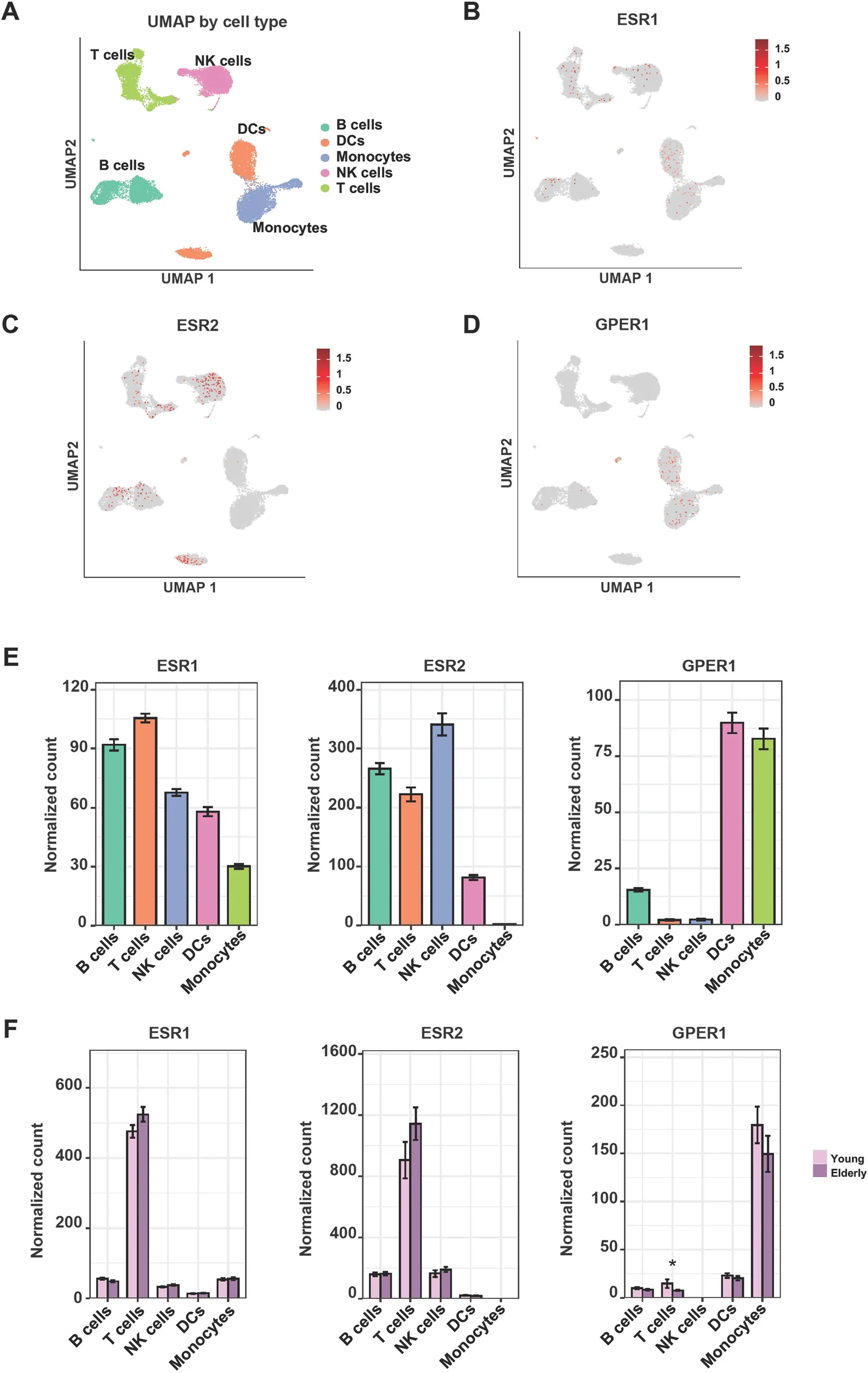
Estrogen receptor gene expression in PBMCs The Sound of Life scRNAseq dataset was re-analyzed to examine the expression of female hormone receptors within PBMC subpopulations, including B cells, DCs, monocytes, NK cells, and T cells. **(A)** UMAP of PBMC subpopulations, generated from 500 subsampled single cells per major cell type from the full cohort (n=96), log-normalized and embedded using Seurat, colored by cell type or expression of *ESR1* **(B)**, *ESR2* **(C)**, and *GPER1* **(D)** across PBMC subpopulations. **(E, F)** Normalized counts were generated from pseudobulk datasets per PBMC subset using DESeq2 median-of-ratios method and plotted using ggplot2: **(E)** includes the full cohort (n=96 subjects), while **(F)** show only young (n=28, in pink) and elderly women (n=27, in purple). DESeq2 was used to perform differential gene expression between young and elderly women for ESR1, ESR2, and GPER1 (*B H adjusted p<0.05).

Our findings show that estrogen receptor expression in immune cells differs by receptor subtype and cellular location. Specifically, cytotoxic cells such as NK and T cells exhibit distinct patterns of ER expression. NK cells show a high membrane expression of ERα and a substantial surface expression of GPER1, while ERβ is minimally expressed and found intracellularly. In contrast, T cells express moderate membrane ERα and lower membrane GPER1 levels, while ERβ is mainly found intracellularly at low levels. In women, T cells show strong ESR1, ESR2, and GPER1 transcript levels compared to NK cells. These results demonstrate distinct ER expression patterns in NK and T cells, highlighting their ability to respond to estrogen signals via membrane ERs, which may influence cytotoxic activity and modulate immune responses.

### Hormonal treatment increases the number of CD3+ cells in irritated skin

Our previous work in post-menopausal women skin showed that myeloid cells, including Langerhans cells, dermal dendritic cells, and macrophages, are influenced by female hormones. Following an SLS challenge, HRT significantly increased the frequency of these skin cell populations compared to those not on HRT (21).To expand on our previous findings, we focused our investigation on changes in T and NK cytotoxic cells in the skin after SLS and HRT treatment, specifically examining their ERα expression. We induced skin irritation by applying 1.25% w/v SLS to occluded buttock skin for 48 hours (21). Using samples from our previous study, we performed immunohistology assessments on SLS-exposed skin from three groups: premenopausal women (pre-M, n=8), post-menopausal women with hormone replacement therapy (post-M+HRT, n=7), and post-menopausal women without hormone replacement therapy (post-M, n=9).

To clarify the immunomodulatory role of HRT, we assessed ERα expression following SLS challenge in pre-menopausal and post-menopausal women, both with and without HRT. There were no significant differences in the percentages of NK (CD56+) or T (CD3+) cells expressing ERα in unchallenged skin among groups (**Figures 3A, B, E**). Subsequently, we quantified cell numbers and observed a significant increase in total T cells in SLS-challenged skin compared to unchallenged skin in pre-menopausal women [pre-M: 1543 ± 518 cells/mm^2^ (unchallenged) vs. 2495 ± 749 cells/mm^2^ (SLS) (P < 0.01)] and in post-menopausal women receiving HRT [post-M+HRT: 2094 ± 1418 cells/mm^2^ (unchallenged) vs. 3539 ± 1663 cells/mm^2^ (SLS) (P < 0.01)]. This increase was not significant in postmenopausal women without HRT (**Figure 3C**).

**Figure 3:**
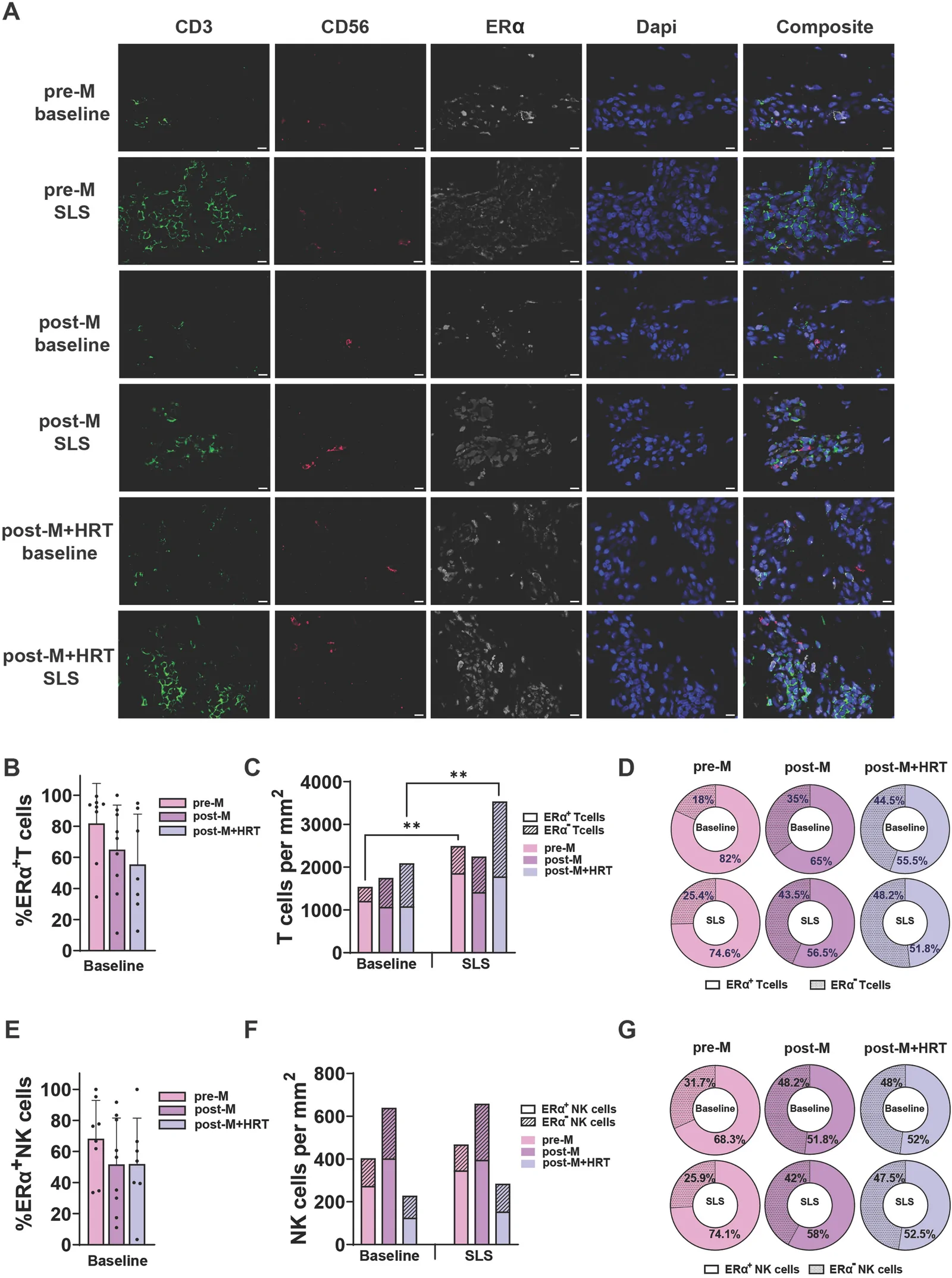
Hormonal treatment increases CD3^+^ T cells in skin after SLS challenge. The presence of T cells (CD3) and NK cells (CD56) in pre- and post-menopausal women was evaluated by immunofluorescence analysis. **(A)** Representative photomicrographs show CD3^+^ cells (green), CD56^+^ cells (red), and ERα^+^ cells (gray) in baseline skin and sodium lauryl sulfate (SLS)–challenged skin from pre-menopausal women [pre-M, n=8)] and post-menopausal women (post-M) with (n=7) or without (n=9) hormone replacement therapy (HRT). T cell and NK cell densities are shown in **(C, F)**, while the percentage of ERα expression within each cell subset is shown in **(B, E)**. The proportions of ERα^+^ and ERα^-^ cells within T-cell and NK-cell populations at baseline and after SLS challenge are shown in **(D, G)**; the dashed pattern represents the ERα^-^ cell population. Data are presented as mean ± standard deviation (SD). Group differences were analyzed using one-way ANOVA followed by Tukey’s multiple-comparisons test (** p>0.01). Scale bar = 20 µm.

Next, we compared ERα^+^ T cell proportions across groups. In unchallenged skin, post-menopausal women had 35% ERα^+^ T cells versus 18% in pre-menopausal women, and 45% in the post-M+HRT. Following SLS exposure, ERα^+^ T cells rose to 25% in pre-menopausal and 43.5% in post-menopausal women, while HRT-treated post-menopausal women showed minimal change (48.5%), this trend is illustrated in **Figures 3C, D**.

NK cell numbers were largely unaffected by SLS challenge in all groups. In unchallenged skin, NK cells tended to be higher in post-menopausal women and lower in post-menopausal women on HRT compared to pre-menopausal women, however the differences were not statistically significant. After SLS, ERα^+^ NK cells decreased slightly in pre-menopausal (31.7% → 25.9%) and post-menopausal women (48.2% → 42%) and remained stable with HRT (48% → 47.5%) with no statistically significant differences (**Figures 3F, G**).

These results indicate that hormonal status selectively influences lymphoid populations during skin inflammation. In post-menopausal women, HRT enhances T cell responses to irritation but does not affect NK cells, suggesting distinct regulatory mechanisms for these subsets.

### Premenopausal hormones selectively modulate IFN-γ production in T and NK cells

After observing that HRT increased T cell numbers in the skin of post-menopausal women exposed to SLS, we investigated whether female hormones affect T and NK cell function by assessing mediator production. Although NK cell numbers were not affected by SLS exposure, we examined NK cells alongside T cells, since they complement the immune cytotoxic functions of T cells. Using an *in vitro* system with PBMCs from healthy donors, we stimulated cells with LPS to replicate inflammatory signals associated with skin barrier damage. The cells were then incubated with female hormone cocktails reflecting pre-menopausal (pre-M) and post-menopausal (post-M) concentrations. We then measured the production of key immune mediators in NK and T cells, focusing on the Th1 cytokines IFN-γ, granzyme B, and perforin. We first assessed cell viability at increasing female hormone levels and observed no effect (**Figure 4A**). Next, we evaluated ERα expression on T and NK cells. While higher hormone doses slightly increased the percentage of ERα+ T cells in the absence of stimulation, LPS treatment significantly increased ERα on the T cell membrane, regardless of hormone exposure (**Figure 4B**). In unstimulated NK cells, ERα expression increased in a dose-dependent manner with female hormones (Ø: 75.6% ± 9, post-M: 64.4% ± 13.4, pre-M: 71.4% ± 15, 10xpre-M: 82.1% ± 14.1). LPS stimulation significantly increased ERα on NK cells only with the post-M and pre-M cocktails, while the 10xpre-M cocktail did not further enhance ERα expression in the presence of LPS (**Figure 4B**).

**Figure 4:**
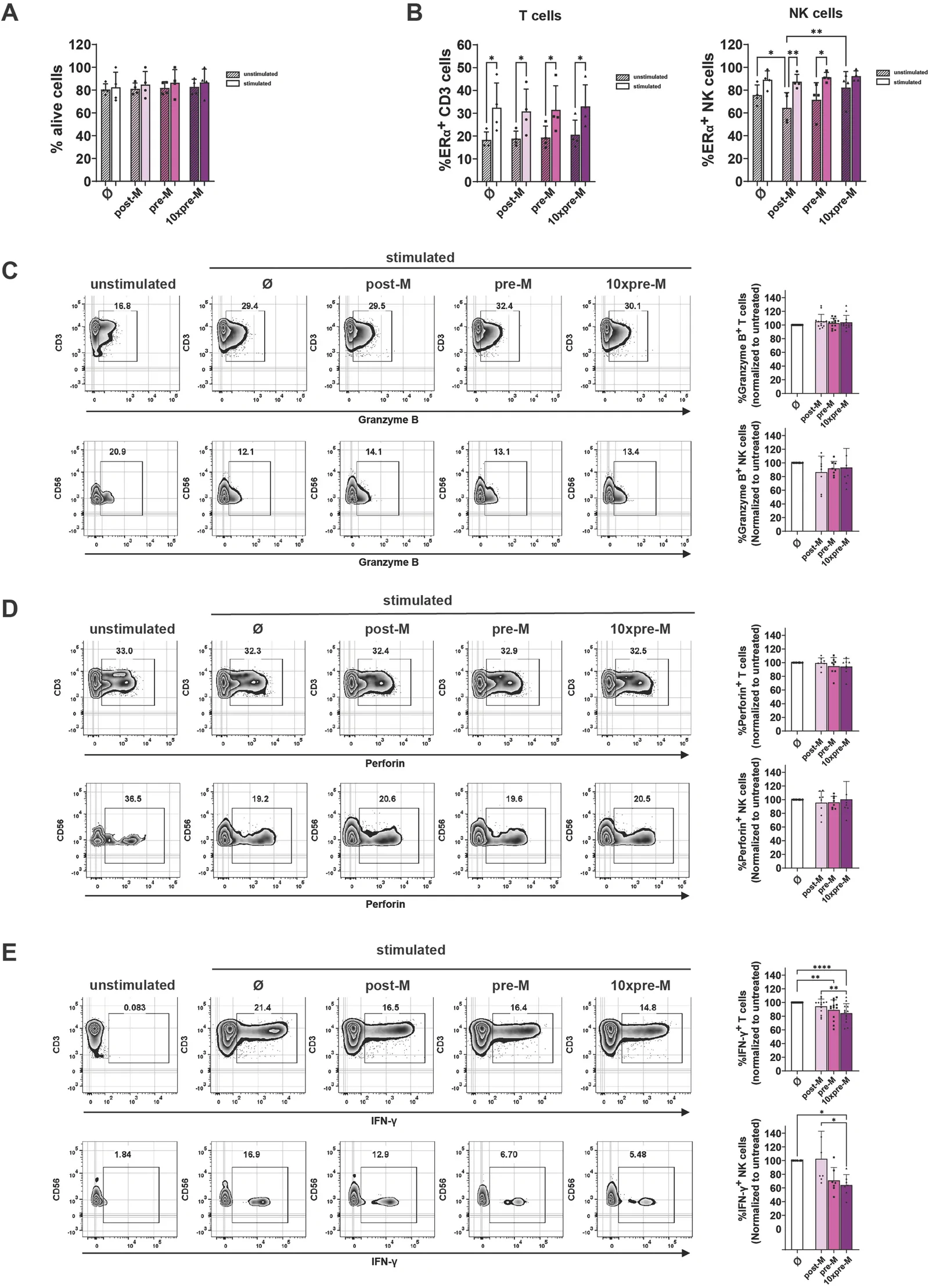
Pre-menopausal hormones reduce IFN-γ production without altering cytotoxic mediators in T and NK cells. PBMCs were incubated with post-menopausal (post-M) pre-menopausal (pre-M), or 10× pre-menopausal hormone (10xpre-M) cocktails and stimulated with lipopolysaccharide (LPS; 100 ng/mL) for 24 h. During the final 4 h, cells were treated with PMA/ionomycin in the presence of brefeldin **(A)** Surface and intracellular staining was performed to assess viability **(A)**, ERα expression **(B)**, and production of granzyme B **(C)**, perforin **(D)**, and IFN-γ **(E)**. Data are shown as mean ± SD (n = 4–13). Statistical analyses were performed using two-way ANOVA **(A, B)** and one-way ANOVA with Tukey’s multiple-comparisons test **(C–E)**. (*p>0.05, ** p>0.01 and ****p>0.0001).

Analysis of intracellular mediator production in stimulated T and NK cells showed that granzyme B (**Figure 4C**) and perforin (**Figure 4D**) levels remained unchanged across hormone conditions. In contrast, IFN-γ production in T cells decreased in a dose-dependent manner with increasing concentrations of female hormones (Ø: 100, post-M: 94.4% ± 10.5, pre-M: 89% ± 15, 10xpre-M: 82.2% ± 14), with significant differences between untreated and pre-M (p=0.0032), untreated and 10xpre-M (p<0.0001), and post-M versus 10xpre-M (p=0.006) (**Figure 4E**). NK cells also showed a dose-dependent reduction in IFN-γ production (Ø: 100, post-M: 102.5% ± 40, pre-M: 70.9% ± 18.9, 10xpre-M: 64% ± 15.5), with significant differences between untreated and 10xpre-M (p=0.023), and post-M and 10xpre-M (p=0.014) (**Figure 4E**).

These results suggest that LPS stimulation enhances surface ERα expression on immune cells, while female hormones selectively downregulate IFN-γ production without affecting cytotoxic mediators. This reduction in IFN-γ may shape skin immune responses in women.

## Discussion

We characterized estrogen receptor expression in peripheral blood immune cell subsets. Supervised gating revealed that ERα was the membrane hormone receptor with the highest cell expression percentage among those analyzed. ERα was predominantly localized to the cell membranes of NKT and NK cells, with lower but notable expression in T cells, and was widely expressed intracellularly among all immune cell types. By contrast, ERβ expression was scarce at the cell surface and was generally lower intracellularly. GPER1, meanwhile, mirrored the intracellular distribution of ERα and was also detected on the membrane of NKT and NK cells. Such distinct localization patterns indicate that individual receptor subtypes may exert specialized regulatory functions within the immune compartment.

Research shows that pre-menopausal women have monocytes with lower ESR1 RNA levels compared to those in men and post-menopausal women, suggesting a correlation between elevated estradiol levels and diminished ESR1 expression. Conversely, no notable differences in ESR1 and ESR2 RNA levels were detected in B and T lymphocytes of both genders, nor in lymphocytes from pre- and post-menopausal women (34). In our investigation, we used PBMCs from donors of unspecified sex *in vitro*, which express ERs and respond to female hormones and exhibited variations in the response to female hormone treatment in T and NK cells. Further studies are necessary to elucidate the mechanisms, particularly regarding sex and menopausal status of individuals involved. We showed that in the presence of the female hormone cocktail, ERα expression in NK cells increases, while T cells show little change. However, both cell subsets exhibit a reduction in IFN-γ production. One possible explanation is that NK and T cells have different ERα expression patterns at baseline, or that different subset dynamics within the T cell population drive the response. To better understand this discrepancy in female hormone response, further analysis on different human T cell subsets, particularly CD4 and CD8, is needed. Upon stimulating T and NK cells, we observed an increase in ERα on the cell membrane, indicating rapid receptor trafficking or isoform redistribution upon activation. This suggests that swift estrogen responses might occur through non-classical, membrane-associated pathways, indicating that these cells are equipped for rapid, non-genomic ERα-dependent modulation of immune responses (38). In immune cells, ER expression can fluctuate with stimulation; certain cytokine/milieu releases can elevate both ESR1 RNA and ERα protein levels. Post-menopausal changes could thus reflect altered milieu-driven receptor regulation, in addition to estradiol levels alone (39), where compensatory upregulation in response to estrogen loss may occur, with cells increasing ESR1 expression when ligand exposure decreases, to maintain sensitivity (40).

We compared SLS-exposed skin from pre- and post-menopausal women, both with and without HRT. HRT restored T-cell counts in menopausal skin to levels seen in pre-menopausal women. Additionally, *in vitro* exposure of PBMCs to female hormone cocktails showed a dose-dependent reduction in IFN-γ production by T and NK cells, whereas cytotoxic mediators, such as granzyme B and perforin, remained unchanged. These findings suggest that female hormones selectively modulate cytokine secretion, reducing pro-inflammatory signaling while maintaining cytotoxic function, thereby supporting immune homeostasis in hormonally influenced skin.

Despite advances in understanding estrogen receptor localization and function, studies on female hormone activities in primary human immune cells remains limited, with most research to date conducted in murine models. While numerous studies have shown that estrogen and its receptors influence the development and maturation of T cells and other immune cells, the overall impact of estrogen on immune function remains debated.

Previous studies have investigated the gene expression of estrogen receptors in sorted T cells, NK cells, monocytes, and B cells in pre-menopausal and post-menopausal individuals using qPCR (33). In line with our scRNAseq findings, these studies have demonstrated that the most abundant estrogen receptor is ERα, while GPER1 expression was observed only in T cells, monocytes, and B cells. No significant differences in ER distribution were detected between cell types or between groups. However, our study reveals differential ER expression across immune subsets. The discrepancy may be attributed to variations in cell isolation techniques and gene expression analysis methods (33). We noticed discrepancies between mRNA and protein expression levels of ESR1, ESR2, and GPER1, as well as their abundance within the immune subset. Several factors, including variations in translation and transcription efficiency, along with differences in assay sensitivity, may contribute to the observed disparity. We also observed inconsistencies between our supervised and unsupervised results, which are attributable to the different analytical methods employed. The supervised gating approach measures the percentage of positive cells for each receptor, identifying cells that exceed an FMO defined expression threshold. In contrast, the unsupervised analysis uses relative signal intensity across all markers, which can bias results toward receptors with high per cell expression even if fewer cells express them or toward receptors with high baseline or nonspecific signals.

Estrogen can modulate T cell subsets (41). It has been shown to enhance IFN-γ secretion by binding ERα to the *IFNG* promoter or by upregulating the Th1 transcription factor T-bet (42–44). One study found increased *IFNG* gene expression during the mid-follicular phase compared to the ovulatory and luteal phases, when estrogen and progesterone levels are higher (33). This suggests that elevated female hormone levels enhance *IFNG* gene expression in PBMCs. However, our data show that, *in vitro*, female hormones decrease IFN-γ production in T and NK cells. This discrepancy may be due to differences in gene and protein expression, or to analyzing single cell types versus pooled PBMCs. Navarro et al. studied IFN-γ and granzyme B production in primary transduced antigen-specific T cells and detected intracellular ERα. When stimulated with a specific antigen in the presence of estrogen, they observed increased production of both cytokines compared to controls. However, the overall percentage of IFN-γ-producing T cells decreased in both females and males as estrogen doses increased, which aligns with our findings using the female cocktail (45). *In vitro* estrogen treatment did not significantly increase ERα or ERβ protein expression in T cells, consistent with our culture results (45).

Although we were unable to quantify progesterone mRNA or protein receptor levels, the absence of detectable progesterone receptors does not necessarily indicate a lack of effect. Progesterone may interact with non-classical, membrane-bound progesterone receptors, which merits further investigation. Nonetheless, progesterone levels also diminish post-menopause, and given that HRT includes progesterone for most women, understanding its role is essential. Progesterone has been shown to promote immune tolerance by inhibiting IFN-γ and perforin secretion in T cells (46, 47). This partially aligns with our findings, except for perforin, which may be due to the presence of estrogen in our hormone cocktail. Additionally, PBMCs from women on HRT produced less IFN-γ than those from women not receiving HRT (48). The effects of HRT on NK cells in menopausal women remain controversial, with studies reporting decreased, increased, or unchanged cytotoxic activity (7, 49, 50). These results highlight the importance of considering not only estrogen or progesterone alone, but both together, as for most women, HRT consists of both estrogen and progesterone.

After SLS-induced skin irritation, T cell numbers increased significantly in both pre- and post-menopausal women receiving HRT, but not in postmenopausal women without HRT. During skin barrier disruption, these changes reflect adaptive immune cell infiltration, which is influenced by hormonal status. The T cell expansion included both ERα-positive and ERα-negative populations, suggesting that endogenous hormones and HRT may promote T cell recruitment, proliferation, or retention in inflamed skin through ERα-independent or indirect mechanisms involving the skin microenvironment. Previous studies have also reported increased T cells and macrophages after SLS (51). HRT has been shown to affect myeloid cell populations, such as Langerhans cells, dermal DCs, and macrophages, following SLS challenge in postmenopausal women (21). These myeloid cells can recruit and activate lymphoid cells, including T cells, through antigen presentation or via soluble mediators such as chemokines and cytokines (52–54). Additionally, fibroblasts and keratinocytes, which are responsive to estrogen (55, 56), can produce mediators that recruit and support T cells after skin barrier damage (57, 58). In contrast, NK cell numbers did not change after SLS challenge, and the literature provides limited data on NK cells in this model. Our model induces mild, acute inflammation, which may not be sufficient to increase NK cell numbers. Overall, our findings in SLS-challenged skin highlight the differential sensitivity of lymphoid populations to female hormones.

A key limitation of this study is the small sample size, which reduces the statistical power to interpret hormone-immune interactions. The *in vitro* hormone concentrations may not fully reflect physiological conditions or individual variability. Additionally, standard cell culture media often contain exogenous estrogens, such as those from fetal bovine serum and the estrogen mimetic phenol red, which can have immediate and long-term epigenetic effects (59). Despite these factors, we still observed the influence of female hormones on lymphoid cells. Future studies may benefit from using hormone-free media to better assess the impact of culture medium components.

A limitation of our study is that the menopausal status of the healthy donor controls was inferred from age rather than confirmed directly (through clinical assessment or hormone level measurement). Although the age ranges selected (25–36 and 55–63 years) fall outside the typical perimenopausal transition window and are consistent with presumed pre- and postmenopausal status, respectively, age-based classification remains an accepted but imperfect proxy in the literature (28, 29), and individual variability in menopause onset means this classification may not precisely reflect the true menopausal status of each donor. Future studies incorporating confirmed menopausal status or hormonal profiling would help validate the findings.

Future research should use longitudinal study designs with larger sample sizes to examine changes in female hormone receptor expression and immune function across hormonal states. Mechanistic studies of ERα and GPER1 signaling pathways are needed to clarify the molecular basis of hormone-driven immune modulation.

GPER1 subcellular localization remains actively debated, with reports of expression at the plasma membrane and in intracellular compartments including the endoplasmic reticulum, Golgi, and early endosomes, with localization varying by cell type, tissue, age, sex, and species. Early studies using GFP-tagged GPER1 found the receptor largely restricted to the endoplasmic reticulum and Golgi rather than the plasma membrane, and this intracellular predominance has since been generally supported in vascular cells, though its functional contribution remains unresolved. Other work has identified additional trafficking routes, such as association with cytokeratin intermediate filaments, alongside canonical membrane signaling including cAMP production and β-arrestin2 recruitment (60, 61). This suggests intracellular GPER1 may represent a functionally distinct signaling pool rather than simply a reserve, with receptor trafficking potentially shaping context-dependent signaling (such as EGFR transactivation, Ca^2+^ mobilization, PI3K/ERK activation).

Our data showed that GPER1 is predominantly expressed intracellularly in B cells, T cells, monocytes, and DCs, while it is mainly present on the cell surface in NK and NKT cells, suggesting distinct functionality.

One limitation of this study is that receptor localization was not examined directly at the single-cell level. Instead, it was inferred from differences in staining between non-permeabilized and permeabilized conditions. This was a useful approach, but it should be interpreted with some caution, as variation in staining efficiency between the two conditions could have affected the apparent balance between surface and intracellular receptor expression. This could be addressed in future work by using imaging flow cytometry, such as ImageStream, on purified immune cell populations. Combining this with ER staining and separate fluorochromes for intracellular and membrane-associated staining would allow receptor localization to be assessed more directly.

Expanding analyses to additional immune cell populations and receptor subtypes, and conducting functional assays of cytotoxicity, antigen presentation, across a range of stimulation conditions, including unstimulated or sub-maximal LPS doses, and migration, will improve clinical understanding. In *in vivo* SLS studies, increasing SLS exposure or concentration could provide a more robust immune challenge.

Clinically, these results highlight the potential of hormone replacement therapy to modulate specific lymphoid populations during skin inflammation, supporting personalized management of inflammatory skin disorders in post-menopausal women. Recognizing receptor subtype-specific effects and hormonal status is essential for optimizing immunomodulatory therapies that leverage hormone-immune interactions while minimizing adverse effects. This work provides a foundation for targeted interventions addressing immune aging and hormone-related skin conditions.

## Data Availability

The datasets presented in this study can be found in online repositories. The names of the repository/repositories and accession number(s) can be found in the article/**Supplementary Material**.

## Supplementary material

Supplementary Figure 1 Supervised gating strategy for flow cytometry. Panel **(A)** Dot plots were generated with side scatter (SSC) against forward scatter (FSC) to isolate cells from debris and aggregates. Singlet cells were gated using FSC-H versus FSC-A, followed by gating of live cells using the live/dead negative gate. Immune cells were identified as CD45+, and B cells were further gated as CD20+CD3-. The CD3+ population was plotted on CD56 versus CD3 to distinguish T cells (CD3+CD56-) from NKT cells (CD56+CD3+). The CD20-CD3- population was plotted on CD14 versus CD56 to identify NK cells (CD56+CD14-). The CD56-CD14 population was plotted on CD14 versus CD16 to gate the monocyte population (CD16+CD14+). The remaining cells were plotted on HLA-DR versus CD11c, where dendritic cells were identified as HLA-DR+. Panel **(B)** shows the relative abundance of B cells, T cells, NKT cells, NK cells, DCs, and monocytes, as determined by supervised analysis.

Supplementary Figure 2 Unsupervised clustering analysis of flow cytometry data. Unsupervised clustering of PBMCs was performed to identify distinct immune cell populations. FlowSOM results are visualized in Uniform Manifold Approximation and Projection (UMAP) space, with Panel **(A)** showing surface staining and Panel **(B)** showing total staining, defined as membrane plus intracellular staining. Panels **(A, B)** display the relative intensity of selected markers across the UMAP space for surface and total staining, respectively. Panels **(C, D)** show heatmaps summarizing the cell populations identified by FlowSOM and their corresponding marker expression intensities. Panel **(E)** shows the relative abundance of B cells, T cells, NKT cells, NK cells, DCs, and monocytes identified by unsupervised analysis. Panel **(F)** shows ERα expression across the different immune subsets identified by unsupervised analysis, while Panels **(G, H)** show ERβ and GPER1 expression, respectively. For ER expression analyses, the median intensity value of membrane expression is shown in dark green bars and total expression in light yellow bars. Data are presented as mean ± standard deviation.
