# Supplementary material for: Female hormones enhance skin T cell expansion and suppress IFN-γ production
Source: Front Immunol. 2026 Oct 1;17:1903552. doi: 10.3389/fimmu.2026.1903552 (PMC13668513; doi:10.3389/fimmu.2026.1903552)
Supplement: Supplementary file 1 [file DataSheet1.pdf]

frontiers

The International Society for the Advancement of Cytometry (ISAC) has highlighted the importance of including comprehensive methodological information to ensure data reproducibility and reliability. In line with this, Frontiers in Immunology now requires authors to submit a checklist for manuscripts that involve flow or mass cytometry. This checklist helps standardize the reporting process, improving the quality and transparency of published data. By doing so, we support scientific progress, making it easier for other researchers to replicate and validate experiments.

This form should be submitted with any manuscripts using flow or mass cytometry.

Sample/specimen/material description

- [ ] Total blood
- [x] PBMCs
- [ ] Organ digests

Other____

Did the samples suffer any treatment before or after incubation with the antibodies?

- [x] Drug Estradiol, Progesterone, DHEA
- [x] Cell permeabilization eBioscience™ Permeabilization Buffer(ThermoFisher Scientific, Cat#00-83
- [x] Dye Zombie UV fixable viability kit
- [ ] Propidium iodine
- [ ] Not applicable

Other BrefeldinA, LPS ,Ionomycin, PMA, human TruStain

Instrument and antibodies

Name of the Cytometer BD FACSymphony and BD LSRFortessa

Antibodies and targets	Fluorochrome/ Metal	Catalog number/Company
e.g. anti-CD4	FITC	Cat. XXX/ XXX ltd.
CD20	BUV805	Cat#612905, BD Berkshire, UK

CD11c	BUV661	Cat#61267, BD
CD3	BV785	Cat#300472, Biolegend,
HLADR	BV711	Cat#367644, Biolegend,
CD45	BV605	Cat#368524, Biolegend,
CD16	BV510	Cat#360730, Biolegend,
CD56	PE-cy7	Cat#318318, Biolegend,
CD14	PE-fire 640	Cat#367154, Biolegend,
ERa	AF488	Cat#AB194150, Abcam
ERb	PE	Cat#AB205541, Abcam
GPER1	coralite plus 647	Cat#PA5-28647,
CD3	PE-cy7	Cat#563423, BD,
CD3	APC-cy7	Cat#300426, Biolegend,
CD56	PE-cy7	Cat#318318, Biolegend,
CD11b	BV711	Cat#740771,BD,
IFNg	AF488	Cat#505813, Biolegend,

Data analyses

1. Name of the software OMIQ and FlowJo v10.8.1
2. Reference gating strategy in the manuscript or supplementary material

Gating strategy in (eg Figure X) supplementary figure 1 and figure 1
