# Supplementary material for: Female hormones enhance skin T cell expansion and suppress IFN-γ production
Source: Front Immunol. 2026 Oct 1;17:1903552. doi: 10.3389/fimmu.2026.1903552 (PMC13668513; doi:10.3389/fimmu.2026.1903552)
Supplement: Supplementary file 2 [file Table1.docx]

**Supplementary Table S1: DESeq2 output for the comparison of ER expression between young and elderly women across immune subsets**

|  | **Gene** | **baseMean** | **log2FoldChange** | **lfcSE** | **stat** | **pvalue** | **padj** | **FC** |
| --- | --- | --- | --- | --- | --- | --- | --- | --- |
| **B cells** | ESR1 | 52.191822 | -0.141062 | 0.102467 | -1.376659 | 0.1686 | 0.5748 | 0.9068 |
|  | GPER1 | 9.319915 | -0.092108 | 0.181933 | -0.506275 | 0.6127 | 0.8816 | 0.9381 |
|  | ESR2 | 160.380505 | 0.00107 | 0.13333 | 0.008023 | 0.9936 | 0.9978 | 1.0007 |
| **DCs** | ESR1 | 13.32086 | 0.208985 | 0.135002 | 1.548013 | 0.1216 | NA | 1.1558 |
|  | GPER1 | 21.69765 | -0.150703 | 0.200148 | -0.752961 | 0.4515 | NA | 0.9008 |
|  | ESR2 | 19.07848 | -0.175064 | 0.196741 | -0.88982 | 0.3736 | NA | 0.8857 |
| **Monocytes** | ESR1 | 54.78326 | 0.093146 | 0.137303 | 0.678402 | 0.4975 | 0.8402 | 1.0667 |
|  | GPER1 | 164.72554 | -0.251364 | 0.225894 | -1.11275 | 0.2658 | 0.7074 | 0.8401 |
| **NK cells** | ESR1 | 34.61123 | 0.214661 | 0.097265 | 2.206977 | **0.0273** | 0.2815 | 1.1604 |
|  | ESR2 | 177.21562 | 0.193144 | 0.212369 | 0.909473 | 0.3631 | 0.7312 | 1.1432 |
| **T cells** | ESR1 | 499.90002 | 0.152222 | 0.078374 | 1.942251 | 0.0521 | 0.1773 | 1.1113 |
|  | ESR2 | 1021.29182 | 0.34039 | 0.206285 | 1.6501 | **0.0989** | 0.2681 | 1.2661 |
|  | GPER1 | 11.33662 | -0.761839 | 0.24252 | -3.141337 | **0.0017** | **0.0169** | 0.5897 |
