# Supplementary material for: Female hormones enhance skin T cell expansion and suppress IFN-γ production
Source: Front Immunol. 2026 Oct 1;17:1903552. doi: 10.3389/fimmu.2026.1903552 (PMC13668513; doi:10.3389/fimmu.2026.1903552)
Supplement: Supplementary file 3 [file Table2.docx]

**Supplementary Table S2: Key resources Table**

| **Reagent and resource** | **Clone** | **Source** | **Identifier** | **Concentration or dilution** |
| --- | --- | --- | --- | --- |
| Zombie UV™ Fixable Viability Kit | N/A | Biolegend, London, UK | Cat#423108 | 1:500 |
| Brefeldin A Solution (1,000X) | N/A | Biolegend, London, UK | Cat#420601 | 1:1000 |
| LPS | N/A | Merck, Dorset, UK | Cat#P8274 | 100 ng/mL |
| Ionomycin | N/A | Merck, Dorset, UK | Cat#I0634 | 1 ug/mL |
| PMA | N/A | Merck, Dorset, UK | Cat#P1585 | 100 nM |
| SLS | N/A | Merck Life Science, Gillingham, UK | Cat#L3771 | 1.25% w/v |
| RPMI 1640 | N/A | Merck, Dorset, UK | Cat#R8758 | N/A |
| FBS | N/A | Life technologies, Paisley, UK | Cat#10500064 | 10% |
| Penicillin-Streptomycin | N/A | Merck, Dorset, UK | Cat#P0781 | 1% |
| eBioscience™ Permeabilization Buffer | N/A | ThermoFisher Scientific, Altrincham, UK | Cat#00-8333-56 | 1% |
| Ficoll Paque Plus |  | Cytvia | Cat#GE17-1440-02 | N/A |
| **Human samples** | | | | |
| Volunteer skin study |  | Dermatopharmacology Unit, Salford Royal NHS Foundation Trust Hospital, Salford, UK | UREC; reference 2017-2954 |  |
| Peripheral blood NC24 leukocyte cones |  | Manchester NHS Blood & Transplant Clinic. | UREC reference 2018-2696-5711 |  |
| **PBMC ER staining (referring to Figure 1)** | | | | |
| **Reagent and resource** | **Clone** | **Source** | **Identifier** | **Concentration or dilution** |
| CD20 BUV805 | 2H7 | BD, Berkshire, UK | Cat#612905 | 1:100 |
| CD11c BUV661 | B-ly6 | BD, Berkshire, UK | Cat#61267 | 1:100 |
| CD3 BV785 | UCHT1 | Biolegend, London, UK | Cat#300472 | 1:100 |
| HLA-DR BV711 | L243 | Biolegend, London, UK | Cat#367644 | 1:100 |
| CD45 BV605 | 2D1 | Biolegend, London, UK | Cat#368524 | 1:100 |
| CD16 BV510 | B73.1 | Biolegend, London, UK | Cat#360730 | 1:100 |
| CD56 PE-cy7 | HCD56 | Biolegend, London, UK | Cat#318318 | 1:100 |
| CD14 PE-Fire 640 | 63D3 | Biolegend, London, UK | Cat#367154 | 1:100 |
| ERα AF488 | E115 | Abcam, Cambridge, UK | Cat#AB194150 | 1:100 |
| ERϐ PE | ERb455 | Abcam, Cambridge, UK | Cat#AB205541 | 1:50 |
| GPER1 (coralite plus 647) | rabbit polyclonal | Invitrogen | Cat#PA5-28647 | 1:50 |
| **IFNg flow staining (referring to Figure 4)** | | | | |
| **Reagent and resource** | **Clone** | **Source** | **Identifier** | **Concentration or dilution** |
| Human TruStain FcX™ (Fc block) | N/A | Biolegend, London, UK | Cat#422302 | 1:100 |
| CD3 PE-cy7 | UCHT1 | BD, Berkshire, UK | Cat#563423 | 1:100 |
| CD3 APC-cy7 | UCHT1 | Biolegend, London, UK | Cat#300426 | 1:100 |
| CD56 PE-cy7 | HCD56 | Biolegend, London, UK | Cat#318318 | 1:100 |
| CD11b BV711 | ICRF44 | BD, Berkshire, UK | Cat#740771 | 1:100 |
| IFNg AF488 | XMG1.2 | Biolegend, London, UK | Cat#505813 | 1:100 |
| **Skin Immunofluorescence staining (referring to Figure 3)** | | | | |
| **Reagent and resource** | **Clone** | **Source** | **Identifier** | **Concentration or dilution** |
| ESR1 | 3556 | Antibodies.com, Cambridge, UK | Cat#A251662 | 1:100 |
| CD56 | goat polyclonal | R&D Systems, Abingdon, UK | Cat#AF2408 | 1:100 |
| CD3 | rabbit polyclonal | Dako, UK | Cat#41758441 | 1:200 |
| Donkey serum | N/A | Abcam, Cambridge, UK | Cat#ab50134 | 5% |
| Donkey anti-Rabbit IgG AF488 | polyclonal | ThermoFisher Scientific, Altrincham, UK | Cat#A-21206 | 1:500 |
| Donkey anti-Mouse IgG AF647 | polyclonal | ThermoFisher Scientific, Altrincham, UK | Cat#A-31571 | 1:500 |
| Donkey anti-Goat IgG AF555 | polyclonal | ThermoFisher Scientific, Altrincham, UK | Cat#A-21432 | 1:500 |
| Fluoromount-G | N/A | Southern Biotech, Birmingham, AL, USA | Cat#0100-01 | N/A |
| **Software** | | | | |
| OMIQ |  | OMIQ | [https://www.omiq.ai](https://www.omiq.ai/) |  |
| GraphPad Prism software version 10.2.3 |  | GraphPad Prism Software | [https://www.graphpad.com](https://www.graphpad.com/) |  |
| FlowJo v10.8.1 |  | BD Life Sciences | [https://www.flowjo.com](https://www.flowjo.com/) |  |
| Zotero |  | Digital Scholar | [https://www.zotero.org](https://www.zotero.org/) |  |
| Grammarly |  | Superhuman Go | [https://app.grammarly.com](https://app.grammarly.com/) |  |
